# The evolving landscape of hybrid viral vector architectures: a persisting niche

**DOI:** 10.3389/fbioe.2026.1900662

**Published:** 2026-10-16

**Authors:** Konstantin Kochergin-Nikitsky, Svetlana Smirnikhina

**Affiliations:** Laboratory of Genome Editing, Research Centre for Medical Genetics, Moscow, Russia

**Keywords:** chimeric vectors, episomal persistence, gene delivery platforms, hybrid viral vectors, packaging capacity, site-specific integration, tropism modulation

## Abstract

The development of hybrid viral vectors was historically driven by the need to overcome inherent limitations of individual viral platforms, including restricted payload capacity, narrow tropism, and limited control over genomic persistence. By integrating functional modules from diverse viral and non-viral systems, these chimeric constructs exploit complementary mechanisms to expand therapeutic potential. This review examines the architectural principles underlying hybrid vector design, framing their evolution as a logical extension of viral engineering strategies - such as pseudotyping and genetic chimerization - rather than as a retrospective response to the rise of non-viral delivery platforms. We highlight key structural configurations, including adenoviral, lentiviral, herpes simplex virus (HSV) amplicon, baculoviral, and bacteriophage-derived chimeras, as well as their integration with transposon systems (e.g., Sleeping Beauty) to enable enhanced persistence and increased cargo capacity. In parallel, we analyze the biological and technological trade-offs associated with increased structural complexity, including manufacturing constraints, cumulative immunogenicity, and regulatory challenges. Collectively, hybrid vector systems represent a modular and evolving design paradigm that complements advances in monoplatform vectors and remains a valuable approach for addressing complex therapeutic challenges, for example those requiring expanded capacity, multifunctionality, or combinatorial control of gene delivery outcomes.

## 1 Introduction

Efficient delivery of genetic material remains the principal limiting factor in gene and nucleic acid therapy. Regardless of cargo type - plasmid DNA, viral genomes, mRNA, or ribonucleoprotein complexes - therapeutic success depends on the vector’s ability to overcome a sequential series of biological barriers: stability in systemic circulation, tissue tropism, cellular entry, endosomal escape, intracellular trafficking, and, when required, nuclear import and sustained gene expression. As evolutionarily shaped self-replicating gene transfer systems, viral vectors possess integrated structural and functional features that actively mediate these steps. Receptor-defined entry, endosomal escape or membrane fusion, microtubule-associated intracellular trafficking, and regulated nuclear delivery are encoded within viral architecture rather than externally engineered. The molecular fate of delivered genomes is therefore determined not only by cargo design but by platform-specific post-entry mechanisms. Active nuclear import through nuclear pores may involve interactions between viral capsids or pre-integration complexes and cellular nucleoporins, as exemplified by lentiviral vectors capable of transducing both dividing and post-mitotic cells (Freed and Martin, 1996; Wyatt and Sodroski, 1998; Reiser, 2000; Cronin et al., 2005; Sun et al., 2010; Kubo et al., 2012; Lindemann et al., 2013; Hastie et al., 2013; Duvergé and Negroni, 2020). Genome processing further differs across platforms, including episomal circularization and concatemerization in AAV-derived systems or chromosomal integration establishing stable proviral structures in retro- and lentiviral vectors (Lieber et al., 1999; Shayakhmetov et al., 2002; Hac et al., 2025).

Since the late twentieth century, gene therapy has been shaped by several dominant viral platforms, including adenoviruses, γ-retroviruses, lentiviruses, and adeno-associated viruses (AAV). Each provided a distinct balance of efficiency and biological constraint. Adenoviral vectors (AdVV) enabled robust transient expression and efficient transduction of non-dividing cells but were associated with pronounced immunogenicity. γ-Retroviruses and lentiviruses supported stable genomic integration and long-term expression, yet raised concerns regarding insertional mutagenesis; γ-retroviruses additionally required cell division for efficient integration. AAV vectors demonstrated a comparatively favorable safety profile and durable expression in post-mitotic tissues but were limited by a restricted packaging capacity (∼4.7 kb), manufacturing challenges, and limited feasibility of re-administration due to neutralizing antibodies (Reiser, 2000; Lieber et al., 1999; Shayakhmetov et al., 2002; Hac et al., 2025; Nowicki et al., 2020; Yoder et al., 2021; Bestor, 2000; Pannell et al., 2000).

By the 1990s, it had become evident that no single viral system simultaneously optimized delivery efficiency, safety, tropism, and genomic payload capacity. Within this context - well before the emergence of contemporary lipid nanoparticle (LNP) platforms - hybrid viral vectors (VV) began to appear as a logical extension of viral engineering. Pseudotyping of retroviral vectors with vesicular stomatitis virus glycoprotein (VSV-G) expanded tropism and enhanced particle stability (Duvergé and Negroni, 2020; Emi et al., 1991; Friedmann and Yee, 1995; Yu and Schaffer, 2006). Adeno–retroviral and adeno–AAV hybrid systems were explored to combine the high transduction efficiency of adenovirus with the integration or persistence properties of other vectors. In some cases, non-viral systems could also serve as donors of integration capability: for example, incorporation of transposon genomic elements (terminal repeats) can also yield hybrid vectors able to integrate genetic cargo into the host genome in the presence of transposase activity in target cells. Chimeric constructs incorporating packaging, regulatory, or capsid elements from different viral systems further illustrated an early strategy of functional complementation. Hybridization thus emerged as an intrinsic evolutionary step within viral vector development rather than as a response to non-viral technologies (Duvergé and Negroni, 2020; Lieber et al., 1999; Müther et al., 2009a; Feng et al., 1997; Caplen et al., 1999; Duisit et al., 1999; Kubo and Mitani, 2003; Kuate et al., 2004; Murphy et al., 2002; Tan et al., 1999; Soifer et al., 2001; Cunningham et al., 2024).

In parallel, non-viral delivery platforms evolved substantially, including polymeric carriers and lipid nanoparticles. LNP systems, in particular, have demonstrated high transfection efficiency *in vitro* and have achieved clinical validation in the context of mRNA therapeutics and vaccination. Their advantages include cell-free manufacturing, compositional modularity, and scalable production processes. However, their biological limitations are systemic rather than purely technological. *In vivo* delivery largely depends on endocytic uptake with incomplete and variable endosomal escape; systemic administration often results in pronounced organ accumulation (notably hepatic enrichment for many LNP formulations); active nuclear import mechanisms for DNA cargo are absent; and expression is predominantly transient. In addition, repeated administration may provoke complement activation or adaptive immune responses (Bohana-Kashtan et al., 2004; Hou et al., 2021; Jung et al., 2022). These constraints reflect the absence of evolved viral mechanisms for membrane fusion, intracellular trafficking, and genome processing, and are not fully mitigated by formulation optimization alone.

Against this background, hybrid vectors acquire renewed conceptual relevance. Virus–virus hybrids - through pseudotyping, chimeric capsids, or recombination of functional domains - enable targeted modulation of tropism, intracellular kinetics, and immunological profile while preserving viral entry and trafficking mechanisms. Virus–non-viral hybrids aim to integrate viral fusogenic or capsid-derived elements into synthetic delivery systems, or to combine viral and non-viral packaging strategies, thereby attempting to merge manufacturing modularity with biologically optimized cellular entry (Cronin et al., 2005; Duvergé and Negroni, 2020; Yu and Schaffer, 2006; Müther et al., 2009a; Kubo and Mitani, 2003; Kuate et al., 2004; Murphy et al., 2002; Zheng et al., 2000; Zhu et al., 2019). A substantial proportion of clinically relevant vector systems incorporate heterologous viral components and can therefore be considered, under the broad definition of hybridization adopted here, as hybrid or related vector architectures. These include pseudotyped retroviral and lentiviral vectors incorporating heterologous envelope proteins, particularly VSV-G, as well as rAAV vectors incorporating genetic elements and capsid components derived from different AAV serotypes, which could be considered a borderline form of hybridization (see Table 1 for examples and references). Nevertheless, while such hybrid or related architectures have reached clinical relevance, their clinical development has largely remained within the boundaries of functional chimerization rather than increasingly complex multicomponent platforms.

**TABLE 1:** Examples of clinically relevant viral vectors incorporating heterologous components.

Product/candidate	Vector type	Application	Status/clinical trials
Kymriah (tisagenlecleucel) (Awasthi et al., 2023; Leahy et al., 2018)	VSV-G-pseudotyped HIV-1-derived, SIN-LV	CAR-T therapy for B-cell malignancies (B-cell precursor ALL, DLBCL, FL)	Approved ELIANA (NCT02435849); JULIET (NCT02445248); ELARA (NCT03568461); BELINDA (NCT03570892)
Breyanzi (lisocabtagene maraleucel) (Abramson et al., 2020; Munshi et al., 2021; Morschhauser et al., 2024; Breyanzi, 2022)		CAR-T therapy for B-cell malignancies (DLBCL, HGBCL, PMBCL, FL, MCL)	EMA-approved; TRANSCEND NHL 001 (NCT02631044), TRANSCEND FL (NCT04245839)
Abecma (idecabtagene vicleucel) (Munshi et al., 2021; Sharma et al., 2022)		BCMA CAR-T therapy for multiple myeloma	FDA-approved; KarMMa (NCT03361748), KarMMa-3 (NCT03651128)
Zynteglo (betibeglogene autotemcel) (Negre et al., 2015; Locatelli et al., 2022; Kwiatkowski et al., 2026; Zynteglo, 2019)		Transfusion-dependent β-thalassemia (TDT)	FDA-approved; EMA authorization withdrawn in 2022; HGB-204 (NCT01745120), HGB-205 (NCT02151526), HGB-207 (NCT02906202), HGB-212 (NCT03207009), LTF-303 (NCT02633943)
Libmeldy/Lenmeldy (atidarsagene autotemcel) (Fumagalli et al., 2022; Fahim et al., 2024; Fumagalli et al., 2025)		Early-onset metachromatic leukodystrophy (MLD)	EMA-approved; NCT01560182; NCT03392987
Skysona (elivaldogene autotemcel) (Eichler et al., 2017; Eichler et al., 2024)		Early active cerebral adrenoleukodystrophy (CALD)	Status and trials: FDA-approved; EU marketing authorization withdrawn in 2021; NCT01896102, NCT03852498, NCT02698579
Lyfgenia (lovotibeglogene autotemcel) (Kanter et al., 2022; Lesmana et al., 2024)		Sickle cell disease (SCD) with a history of vaso-occlusive events	Status and trials: FDA-approved; HGB-206 (NCT02140554)
Yescarta (axicabtagene ciloleucel) (Neelapu et al., 2017; Jacobson et al., 2022; Locke et al., 2022)	GaLV-pseudotyped γ-retroviral vector	CAR-T therapy B-cell malignancies (large B-cell lymphoma (LBCL), follicular lymphoma (FL)	Approved; pivotal ZUMA-1 NCT02348216; subsequent ZUMA studies for additional indications
Tecartus (brexucabtagene autoleucel) (Brexucabtagene autoleucel for mantle cell, 2023; Wang et al., 2020)	GaLV-pseudotyped MSCV-based γ-retroviral vector	B-cell malignancies (MCL, B-ALL)	Approved; pivotal ZUMA-2 NCT02601313 and ZUMA-3 NCT02614066
Zolgensma (onasemnogene abeparvovec) (Mendell et al., 2021; Day et al., 2021; Mercuri et al., 2021)	rAAV2/9 (Rep2/Cap9)	SMN1 gene therapy for spinal muscular atrophy (SMN)	FDA-approved; initial U.S. approval 2019; START (NCT02122952), STR1VE-US (NCT03306277), STR1VE-EU (NCT03461289)
Elevidys (delandistrogene moxeparvovec-rokl) (Mendell et al., 2020; Mendell et al., 2025; Mendell et al., 2026)	rAAV2/rh74	Gene therapy for Duchenne muscular dystrophy (DMD)	FDA-approved; currently indicated for patients aged ≥4 years with confirmed DMD mutation; SRP-9001-101 (NCT03375164), ENDEAVOR (NCT04626674), EMBARK (NCT05096221)
Giroctocogene fitelparvovec (PF-07055480/*formerly*, SB-525) (Leavitt et al., 2024)	rAAV2/6 (Rep2/Cap6)	FVIII gene therapy for hemophilia A	Development discontinued after ALTA (NCT03061201) and AFFINE (NCT04370054)
SPK-8011 (dirloctocogene samoparvovec) (Frey et al., 2025)	rAAV with SPK200/LK03 bioengineered capsid		Development discontinued; Phase I/II completed; long-term follow-up ongoing (NCT03003533, NCT03432520). Phase III withdrawn before enrollment
FLT180a (verbrinacogene setparvovec) (Chowdary et al., 2022)	rAAV with synthetic AAVS3 capsid	FIX gene therapy for hemophilia B	Phase I/II completed; development discontinued; not approved. B-AMAZE: NCT03369444; long-term follow-up: NCT03641703; B-LIEVE: NCT05164471

Several factors contribute to this limited translation: architectural and manufacturing complexity (e.g., in one such hybrid system, a Rep-encoding plasmid was chemically conjugated to mature AdV/AAV hybrid particles, devoid of Rep protein, via a polylysine bridge to circumvent known Rep-mediated inhibition of vector production) (Shayakhmetov et al., 2002; Fisher et al., 1996); complex immunological profiles resulting from the combination of viral modules; regulatory intricacies associated with the complex composition of novel chimeric constructs (Quality, preclinical and clinical aspects of gene therapy medicinal products - scientific guideline, 2001); and continuous optimization of “pure” platforms - particularly AAV - through directed evolution and capsid engineering, which partially reduces the perceived need for more complex hybrid designs (Hac et al., 2025; Müther et al., 2009a). The emergence of new therapeutic applications, including the delivery of genome-editing systems, efficient *in vivo* transduction of immune cells, traversal of central nervous system barriers, and tunable duration of gene expression, creates potential areas of application for hybrid delivery systems. Combining complementary functions from different platforms may offer advantages that cannot be achieved by any single platform. (Hac et al., 2025; Yoder et al., 2021; Müther et al., 2009a; Tuerxunyiming et al., 2026; Baek et al., 2026).

This review examines the historical development of hybrid viral vectors, proposes a structural classification into virus–virus and virus–non-viral architectures, analyzes molecular design principles, and discusses translational bottlenecks and future perspectives for hybrid systems to provide measurable functional advantages over existing delivery platforms. In this review, we define hybrid viral vector systems as genome-containing particles in which a virus-derived component forms the core and serves as the principal determinant of function, while the other components are derived either from other viruses or, in rare instances, from non-viral sources. Thus, to keep the scope as focused as possible, we exclude from the discussion other complex entities, such as RNP-packaging VLPs and those based primarily on non-viral delivery systems, such as LNPs.

## 2 Camouflaged viral vectors

The earliest examples of hybrid viral platforms, in a broad sense, are pseudotyped retroviral vectors, including lentiviral ones. Already in the mid-1970s, exogenous surface glycoproteins from other enveloped viruses were incorporated into retrovirus-based vectors (Fisher et al., 1996; Huang et al., 1973; Love and Weiss, 1974; Livingston et al., 1976; Weiss et al., 1977). The rationale was the lack of natural retrovirus serotypes with defined tropism for different host tissues. Modifying the native Env complex (SU/TM) to alter tropism is difficult and usually disrupts fusogenicity; therefore, pseudotyping - replacing the native Env with heterologous envelope proteins - has become a widely used strategy (Wyatt and Sodroski, 1998; Kubo et al., 2012; Lindemann et al., 2013; Dalgleish et al., 1984; Deng et al., 1996; Nguyen and Hildreth, 2000).

Pseudotyping exploits retrovirus budding from plasma membrane lipid rafts. Heterologous envelope proteins, when acylated (e.g., palmitoylated), can be targeted to rafts and incorporated into budding particles (Freed and Martin, 1996; Duvergé and Negroni, 2020; Bohana-Kashtan et al., 2004; Wills et al., 1991; Varior-Krishnan et al., 1994; Saifuddin et al., 1997; Brown and London, 2000; Wyma et al., 2000; Li et al., 2002; Zacharias et al., 2002; Zhang et al., 2003; Gladnikoff and Rousso, 2008; Miyagawa-Yamaguchi et al., 2014; Komura et al., 2016; Sengupta et al., 2019).

VSV-G is the most broadly used pseudotyping envelope. It engages LDLR family receptors, triggers clathrin-mediated endocytosis, and undergoes pH-dependent fusion in endosomes, conferring near-universal tropism and enabling efficient transduction of primary T cells for CAR-T generation (Tuerxunyiming et al., 2026; Baek et al., 2026). VSV-G also increases particle stability, allowing concentration to 10^8^–10^9^ IU/mL compared to 10^6^–10^7^ IU/mL for native MLV vectors (Reiser, 2000; Friedmann and Yee, 1995; Ichim and Wells, 2011; Kim and Lim, 2017; Dautzenberg et al., 2021). Limitations include excessively broad tropism, susceptibility to complement and natural IgM antibodies, and enhanced liver uptake (Cronin et al., 2005; Sun et al., 2010; Hastie et al., 2013; Emi et al., 1991; Yu and Schaffer, 2006; Yee et al., 1994; Ci et al., 2018; Elegheert et al., 2018; Finkelshtein et al., 2013).

Rabies virus (RV-G) provides an alternative: it interacts with neuronal receptors (nAChR, NCAM, p75NTR, and transferrin receptor 1/TfR1), conferring strictly neuronal tropism and retrograde axonal transport, though blood–brain barrier crossing is limited (Duvergé and Negroni, 2020; Thoulouze et al., 1998; Tuffereau et al., 1998; Tuffereau et al., 2007; Hislop et al., 2014; Wang et al., 2023).

A key limitation of rhabdovirus G proteins is that receptor binding and membrane fusion reside in the same molecule. Alphavirus envelopes (E2 for receptor binding, E1 for pH-dependent fusion) separate these functions, allowing independent modulation of tropism via E2 modification. Pseudotyped vectors with E2/E1 from Sindbis (SINV), Chikungunya (CHIKV) or Ross River (RRV) viruses confer broad but distinct tropism profiles. E2 can be engineered: insertion of targeting ligands (e.g., ZZ domain) redirects tropism, and point mutations (e.g., K200R in CHIKV, K251R in RRV) enhance *in vivo* stability and dissemination (Duvergé and Negroni, 2020; Li et al., 2002; Zacharias et al., 2002; Zhang et al., 2003).

A similar segregation of receptor-binding and fusion functions exists in paramyxoviruses (measles virus H/F, Nipah virus G/F). Pseudotyped lentiviral vectors with modified H or G proteins (e.g., H mutations blocking CD46/SLAM, insertion of scFvs or DARPins; truncated GcΔ33/Δ34 forms for Nipah) enable full selectivity for cell surface markers such as CD20, CD8 or EpCAM, with titers for NiV-pseudotyped vectors an order of magnitude higher than for measles-based systems and minimal risk of pre-existing immunity.

Baculovirus GP64 offers a more restricted tropism (limited transduction of hematopoietic cells, preference for liver sinusoidal endothelial cells) and may be less susceptible to complement inactivation (Schauber et al., 2004; Guibinga and Friedmann, 2005; Milani et al., 2024; Matsui et al., 2011; Kataoka et al., 2012). LCMV glycoprotein allows efficient targeting of α-dystroglycan-positive cells, including dendritic cells and immune subsets (Dylla et al., 2011; Zhang et al., 2014; Nousiainen et al., 2026).

Chimerization with orthomyxoviruses (influenza HA/NA) produces replication-defective particles that model influenza entry under BSL-2 conditions. These pseudotypes are valuable for high-throughput screening of antiviral compounds, analysis of neutralizing antibodies, and serological assays (McKay et al., 2006; Hatziioannou et al., 1998; Dong et al., 1992; Haselhorst et al., 2008; Zhang et al., 2008; Su et al., 2008; Ao et al., 2008; Wang et al., 2009; Wang et al., 2008; Wang et al., 2010; Du et al., 2010; Labrosse et al., 2010; Lin and Cannon, 2002; Szécsi et al., 2006; Temperton et al., 2007; Nefkens et al., 2007; Garcia et al., 2010; Ferrara et al., 2021).

Finally, similar pseudotype-based approaches have been developed for neutralization assays and entry studies of rabies/lyssaviruses (Wright et al., 2008; Wright et al., 2009), Ebola virus (GP, tropism via lectins and NPC1) (Wool-Lewis and Bates, 1998; Eichler et al., 2021), coronaviruses (Eichler et al., 2021; Giroglou et al., 2004; Temperton et al., 2005; Fukushi et al., 2006), dengue (Fukushi et al., 2006), hepatitis C virus (E1/E2, hepatocyte tropism via CD81, SR-BI, CLDN1, OCLN) (Hsu et al., 2003; Soares et al., 2019) and others (Ogino et al., 2003; Tamin et al., 2009; Mirow et al., 2021).

## 3 Genetically chimeric VVs

In comparison to pseudotyped viral vectors, whose “hybrid” nature is attributed to the presence of exogenous glycoprotein components, in the hybrid vectors considered here chimerization between viral (and in some cases non-viral) platforms is implemented at the level of the vector genome, with the aim of integrating their functional advantages and overcoming the limitations of the parental systems in gene therapy and bioengineering applications. Such approaches were expected to enable modulation of both tropism and particle stability, packaging efficiency, and transgene expression. At the same time, these systems are characterized by a high level of design complexity and the need to ensure functional compatibility of components, and are currently generally regarded not as universal tools but as specialized platforms for specific applications.

### 3.1 Ad-based hybrids

Adenoviruses (90–100nm, non-enveloped) have a broad host range; human Ad belong to Mastadenoviridae. Their icosahedral capsid contains 28–38 kb DNA with inverted terminal repeats (ITR). Tropism is determined by trimeric L-gene fibers (shaft + knob), binding CAR, CD46, desmoglein-2, and sialic acids (sometimes dual specificity). In host cells, Ad and vectors persist mainly episomally, with low integration (10^−4^–10^−6^/genome). Recombinant Ad vectors (rAdV) are usually replication-deficient; helper-dependent HDAdV devoid of most viral genes and have the largest capacity (up to 37 kb) (Lieber et al., 1999; Shayakhmetov et al., 2002; Hac et al., 2025; Genus, 2026).

#### 3.1.1 Hybrid vectors for episomal persistence

The most prominent restrictions of Ad-based vectors are their immunogenicity and transient persistence in the host cells. The latter could be modulated by changing their intracellular fate creating chimeric vectors. The relatively high packaging capacity of adenovirus capsids can be exploited to generate structurally simple chimeric viral vectors containing only a transgene expression cassette packaged into an adenovirus capsid (Figure 1). This strategy was employed by Lieber et al., aiming to obtain vectors that integrate randomly. Their approach involved replacing the E1 gene in the adenovirus genome with a transgene cassette flanked by AAV ITRs. In producer cells (HEK 293), the double AAV ITRs caused instability and spontaneous deletional recombination, producing dsDNA ΔAd.AAV fragments, including circular ones and fragments with a single AAV ITR. These fragments were then packaged into Ad capsids produced by the cells due to the random retention of the adenovirus ψ-region during recombination. This approach allowed packaging of up to 5.5 kb into a single capsid (up to ∼11.5 kb using a scheme splitting the cassette across two adenoviruses and reassembling it in producer cells via recombination). The yield of target vectors was predictably only a fraction of the total number of packaged particles (usually no more than 1/10) and approximately 10^3^–10^4^ per cell. However, purification by cesium gradient centrifugation allowed titers of 10^12^ vector genomes (VG) per ml (Lieber et al., 1999; Shayakhmetov et al., 2002). The obvious limitation of this approach was the nonspecific and highly improbable integration of such vectors in the absence of the REP protein, which mediates specific integration of wild-type AAV, as well as paired AAV ITRs. Without REP most vector genomes will persist episomally. Nevertheless, the authors suggested a mechanism of vector genome concatemerization, which could, among other things, ensure ITR pairing and the generation of vector genomes of sizes compatible with adenovirus capsids.

**FIGURE 1:**
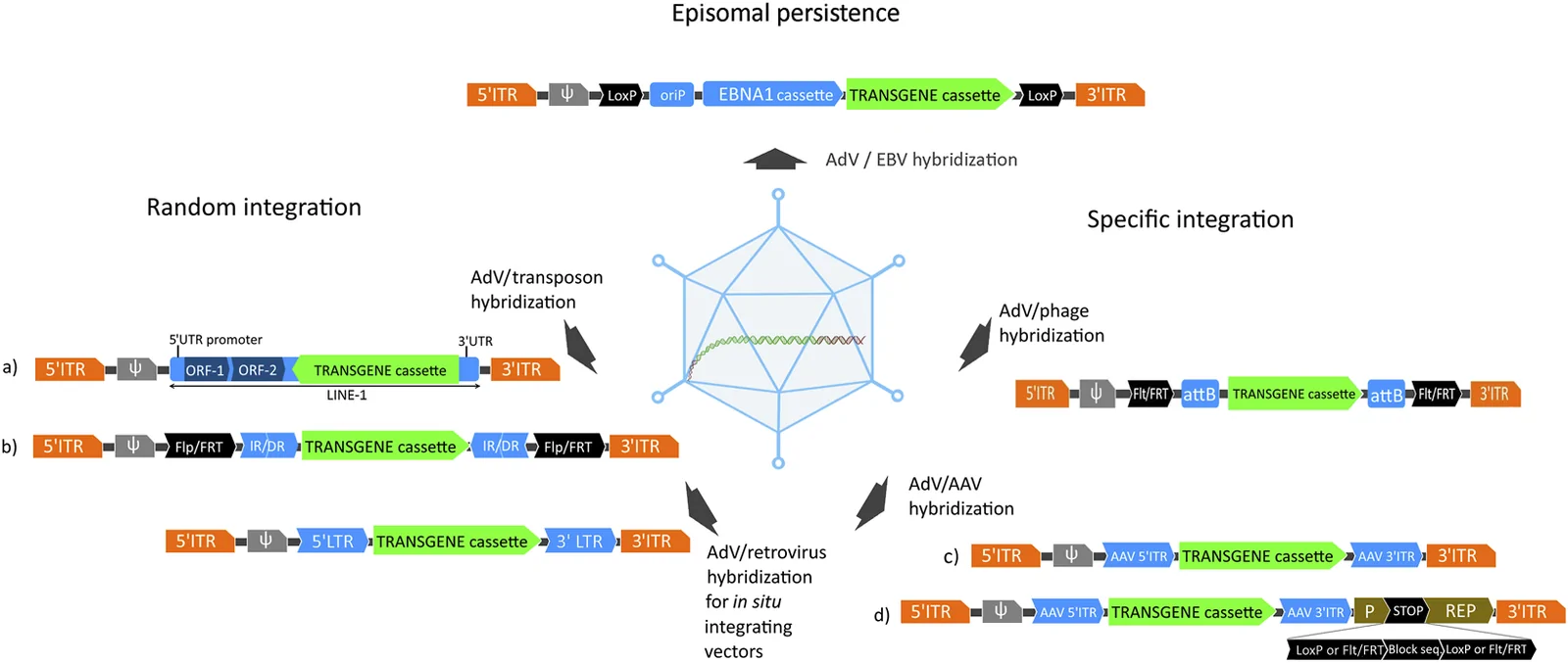
Examples of adenovirus vector (AdV)-based hybrid systems for episomal persistence, random, and site-specific integration. Episomal persistence: genome of the AdV/EBV (Epstein-Barr Virus) hybrid with oriP (cis-acting origin of replication from EBV) and EBNA1 gene for extrachromosomal maintenance. Random integration: **(a)** hybrid with LINE-1 retrotransposon for target-primed reverse transcription (TPRT) based integration mediated by ORF-1/ORF-2 proteins; **(b)** Sleeping Beauty (SB) transposon-based hybrid system with additional Flp/FRT sites for excision/circularization and IR/DR sites serving as substrates for cut-and-paste transposition via transposase. The third construct represents an adenovirus–retrovirus hybrid in which the transgene cassette is flanked by retroviral LTRs, with AdV ITRs positioned outside the LTRs. Specific integration: phage-AdV hybrids with attB sites - substrates for site-specific integration by a phage integrase, and AdV/AAV hybrids without the rep gene **(c)** or with its controlled expression **(d)**. Created with BioRender.com

Hybrid system aiming at stable long-term episomal persistence of the vector genome in the target cells, including dividing ones, was developed by incorporating the EBV episomal maintenance cassette (oriP/EBNA1) into the AdV genome and flanked with LoxP sites. These sites are used by the trans-supplied Cre recombinase to produce circular EBV-like episomes, persisting protractedly in the transduced cells (Tan et al., 1999; Gil et al., 2010). The main limitations of the early versions of this system were high adenovirus-associated immunogenicity (partially solvable by the usage of helper-dependent AdV), double-vector associated complexity and inefficiency, high MOI needed for somewhat efficient double transduction.

#### 3.1.2 Randomly integrating AdV-based hybrids

Alternative strategies for generating integrating (non-specific) Ad-based vectors via hybridization with transposition systems have been developed (Figures 1a,b). The Sleeping Beauty system has been used, in which integration into the genome is mediated by the SB transposase, implementing a characteristic cut-and-paste mechanism (following excision of the transposon from the donor molecule via transposase-recognized terminal repeats (IR/DR) flanking the cargo) into TA dinucleotides of the genome without formation of replication-competent copies (Sleeping Beauty functions as a strictly non-replicative system, mobilized exclusively through excision and reintegration) (Yant et al., 2002; Boehme et al., 2016). Hyperactive variants of the transposase have been developed, including SB11, HSB5, and especially SB100X, which significantly increase transposition efficiency and thereby enhance stable transgene integration (Geurts et al., 2003; Ivics et al., 2007; Mátés et al., 2009). A circular DNA molecule represents an efficient donor/substrate for the SB transposase. The required conversion of the adenoviral genome segment was achieved using the site-specific recombination system Flp/FRT (Flp excises the adenoviral DNA fragment carrying the transposon, additionally flanked by short FRT sequences, resulting in circularization of the excised fragment), after which the SB transposase mediates its integration into the host cell genome (Boehme et al., 2016). Transient expression of the transposase in target cells reduces the risk of uncontrolled remobilization of the transposon, although it cannot be completely excluded. The main limitations of the system include the requirement for co-delivery of two vectors into the same target cells, which increases complexity and reduces overall efficiency (especially *in vivo*), in part due to incomplete processing of the donor substrate and losses at the stages of excision, circularization, and integration, as well as the quasi-random nature of integration without pronounced preferences for specific genomic sites or regions.

Hybrid adenoviral vectors have also been developed using retrotransposon LINE-1 (L1) elements, in which the cargo is incorporated within the L1 retrotransposon construct enclosed in a packaging-competent adenoviral genome, enabling integration via a reverse transcription step. In this system, retrotransposon-encoded proteins (ORF1p/ORF2p) mediate the formation of a ribonucleoprotein complex and its integration via the target-primed reverse transcription mechanism, allowing stable long-term maintenance of transgene expression, including in non-dividing or slowly dividing cells (Soifer et al., 2001; Soifer and Kasahara, 2004; Kubo et al., 2006).

The limitations of the system include poor control of integration sites with the formation of multiple *de novo* insertion copies and, potentially, an increased risk of genomic instability due to the quasi-random nature of retrotransposition.

#### 3.1.3 Adeno–/retro-(lenti-)virus hybrids for random integration

Retroviruses (including lentiviruses), capable of highly efficient integration into the host cell genome, are obvious candidates for the development of hybrid viral vectors capable of long-term expression due to their integration activity. Indeed, in the late 1990s–2000s, studies were published describing the construction of adenovirus/retrovirus hybrid vectors in which the adenoviral component provided efficient delivery to target cells, including difficult-to-transduce and non-dividing cells, while the retroviral component ensured stable integration of the transgene (Figure 1).

Early studies in this field demonstrated the conceptual feasibility of such hybrids, which contained within the genome of an adenoviral vector an LTR-flanked genetic cassette of a replication-defective retroviral vector, enabling the temporary conversion of transduced cells into “factories” producing RVVs that transduced neighboring cells. The fact that the GAG/POL/ENV proteins required for RVV assembly were provided in trans as part of the adenoviral component of the hybrid system and were expressed only transiently ensured the temporary nature of RVV particle production. These constructs were developed with a view toward potential anticancer applications. Modestly efficient transduction of glioma and melanoma cells in mice and rats was demonstrated (10%–15% rate of transduction), as well as stable and long-term expression of transgenes in tumor tissues (Feng et al., 1997; Caplen et al., 1999). In some works (Duisit et al. (Duisit et al., 1999), Kubo and Mitani (Kubo and Mitani, 2003), Kuate et al. (Kuate et al., 2004)) this scheme was refined by introducing TET-regulated control of the packaging proteins expression (VSV-G, used for the formation of the envelope of secondary VV particles, or both VSV-G and GAG/POL) and, consequently, regulation of their production. The use of a lentiviral platform in the latter two studies, compared to retroviral components used by Feng et al. and Caplen et al., provided an advantage in the transduction of non-dividing cells.

Another branch of chimeric Ad/RV vectors was developed with a focus on *in situ* integration. In this case, the construct consisted of a retroviral or lentiviral vector genome retaining only essential elements for integration, inserted into the adenoviral vector. In one of the earliest studies of this type (Zheng et al. (Zheng et al., 2000)), only the LTRs flanking the cargo were essentially retained from the MoMLV retroviral vector; this “cassette” was inserted into a first-generation Ad5-based adenoviral vector. It was assumed that the presence of LTRs would allow the transgene to integrate with some probability, and the authors reported an integration efficiency of approximately 10%–15% (the proportion of cells showing a positive FISH signal after 10 passages). These estimates of integration efficiency, however, should be interpreted with caution given the absence of retroviral integrase and, consequently, of a canonical integration mechanism. In later studies, integration-relevant retroviral components were either incorporated into the hybrid vector, particularly retroviral integrase, or supplied in trans. In the vector developed by Murphy et al., in 2002, the retroviral cassette was additionally flanked by LoxP sites to allow its excision from the AdV backbone, under the assumption that the resulting circular DNA forms would provide better accessibility of the LTRs for integrase (INT)-mediated integration. However, this did not lead to high integration efficiency, which remained 10–50-fold lower than that of native retroviral systems. According to the authors’ interpretation, this was likely due to the lower suitability of circular DNA molecules as substrates for INT compared to linear double-stranded DNA (Murphy et al., 2002).

The development of adenovirus/foamy virus (Ad/FV) hybrid vectors appeared particularly promising. A key advantage of such systems is their relative biosafety, as Foamy virus (spumaviruses) have not been associated with any clinically significant pathology in either their natural hosts or infected humans. In addition, they exhibit a more favorable integration profile, characterized by weaker preferences with respect to regulatory genomic regions - as observed for many γ-retroviruses - or actively transcribed genes, as is typical for lentiviruses (Lindemann and Rethwilm, 2011). Both conceptual implementations were explored: one based on the “producer cell” paradigm, in which primary target cells transiently generate viral vectors, and another relying on direct *in situ* integration of the delivered genetic material (Picard-Maureau et al., 2004; Russell et al., 2004). In both cases, the retroviral component of the hybrid genome was reduced to the minimal cis-acting sequences required for integration along with the transgene cassette, while all other genes required for packaging (in the “vector factory” setting) and integration were supplied in trans - either via separate adenoviral vectors or as independent expression cassettes within a single helper-dependent adenoviral vector carrying the foamy virus module.

The resulting vector titers in producer systems were broadly comparable, on the order of 10^9^–10^10^ per ml. However, while the single-genome hybrid design achieved up to 70% transduction efficiency in secondary target cells (Picard-Maureau et al., 2004), the three-vector system performed substantially worse, largely due to low titers of secondary retroviral particles (approximately 10^3^ per ml) (Russell et al., 2004). Integration efficiency remained modest, reaching only 5%–10% of cells at multiplicities of infection (MOI) in the range of 20–100.

The main studies related to Ad/RV hybridization were conducted in the 2000s. After 2010, only a few isolated publications appeared. Thus, building on the concepts of Soifer et al. (Soifer et al., 2002) and exploiting the advantages of HDAd vectors, S. Kubo and colleagues in 2011 proposed a system for the delivery of a full-length replication-competent retroviral vector (RCR) encoding a therapeutic gene – yeast cytosine deaminase, which converts the non-toxic prodrug 5-fluorocytosine into the cytotoxic 5-fluorouracil – for the treatment of orthotopic tumors (glioma, mesothelioma). Strategically, the authors aimed to address the limited efficiency of retroviral penetration into tumors: the adenovirus, owing to its high transduction efficiency, serves as an initial platform, establishing primary sites of RCR production directly within the tumor, which leads to lateral spread of the retroviral vector and a pronounced antitumor effect. Despite impressive tumor eradication results in mice, the system carries fundamental safety concerns related to uncontrolled dissemination of the RCR in the organism and the associated risk of insertional mutagenesis (Kubo et al., 2011).

#### 3.1.4 AdV-based hybrids for site-specific or semi-specific integration

During the development of Ad-based hybrid vectors consistent with this paradigm, an approach based on the site-specific recombination system of the bacteriophage PhiC31 integrase was employed, enabling semi-specific integration through recognition of a limited set of genomic “pseudo-attP” sites (Thorpe and Smith, 1998; Ehrhardt et al., 2007) (Figure 1). In mammalian cells, PhiC31 integrase is capable of utilizing endogenous sequences resembling attP, thereby catalyzing unidirectional integration into a restricted number of preferred genomic loci (Thorpe and Smith, 1998; Thor et al., 2000). The overall design in this case is broadly similar to that described above for hybrid systems based on the Sleeping Beauty transposase, including a two-vector configuration and, in some implementations, the use of Flp/FRT-mediated circularization of the expression cassette prior to integration. The system exhibits a bias of integration events toward a limited set of frequently used sites (so-called ‘hot spots’, such as mpsL1 in the murine liver), which has been considered a potential factor contributing to a reduced risk of insertional mutagenesis, despite the absence of strict site specificity (Chalberg et al., 2006; Aneja et al., 2007).

One distinct direction in integrating Ad/AVV hybrids development is the exploitation of the Rep-mediated integration to ensure relatively safe and long-term expression. One of the earliest efforts in this area was the work of Fisher et al. (Fisher et al., 1996). A hybrid Ad/AAV vector was constructed, in which E1 was replaced by a transgene cassette flanked by AAV ITRs. To enable the system to function, the authors electrostatically attached a Rep-encoding plasmid to the virions via a poly-lysine linker fused to the capsids, allowing the plasmid and virions to enter target cells together. The authors demonstrated rescue and replication of the AAV genome in cells in the presence of a helper adenovirus. The main drawbacks of the system were high *in vivo* immunogenicity due to adenoviral protein expression, Rep-dependent cytotoxicity, and the overall complexity and instability of the system, which also negatively affected its cost.

Rep protein exerts cytotoxic effects on cells and interferes with efficient production of adenoviral vectors, including hybrid ones. To mitigate this, its expression has been restricted by various strategies. In 1999, Recchia et al. (Recchia et al., 1999) employed a system based on two helper-dependent adenoviral vectors. One vector carried an expression cassette flanked by AAV ITRs, while the other encoded the rep78/68 genes under the control of weak tissue-specific promoters, which reduced interference with vector production. In the transduced target cells Rep expression was also transient yet sufficient to mediate site-specific integration of the rAAV cassette delivered by the hybrid vector. The use of helper-dependent AdVs also reduced immunogenicity. Integration of the transgene into the AAVS1 locus on human chromosome 19 was observed in up to ∼40% of integration events.

Further improvements aimed at regulating Rep expression were subsequently introduced. Ueno et al., in 2000 employed a Cre/loxP-dependent system for irreversible activation of Rep expression, in which a Cre recombinase cassette encoded within the hybrid vector (but outside the ITR-flanked integratable cassette) excised a blocking sequence that prevented Rep expression (Ueno et al., 2000). Similar system with Flp-recombinase-dependent REP-repressor inactivation was proposed by Goncalves et al., in 2008 (Gonçalves et al., 2008). In turn, Recchia et al., in 2004 proposed tetracycline-activation based system (Recchia et al., 2004).

### 3.2 Retroviral vectors with hybrid integrating strategy

A defining property of retroviral vectors is their ability to integrate genetic material into the host genome, a process mediated by viral integrase following reverse transcription of the RNA genome. Although this enables stable transgene expression (transcriptional silencing of integrated proviral sequences is also well documented and can significantly affect long-term expression (Bestor, 2000; Pannell et al., 2000; Nowicki et al., 2020)), integration is not random and exhibits virus-specific preferences for genomic features such as transcriptionally active regions or promoter-proximal sites (the latter being characteristic particularly for γ-retroviruses such as MLV, whereas lentiviral vectors more frequently target actively transcribed gene bodies). Integration site selection is influenced by host factors that tether retroviral preintegration complexes to chromatin, resulting in non-random integration profiles (Yoder et al., 2021). These biases are associated with genotoxic risk.

To improve safety and achieve better control over insertional events, different engineering strategies have been developed to modify or replace the native viral integration machinery. Roughly these approaches could be distributed into two separate groups: (1) re-engineering of the viral integrase and (2) full decoupling of the integration process with substitution by external systems (Figure 2).

**FIGURE 2:**
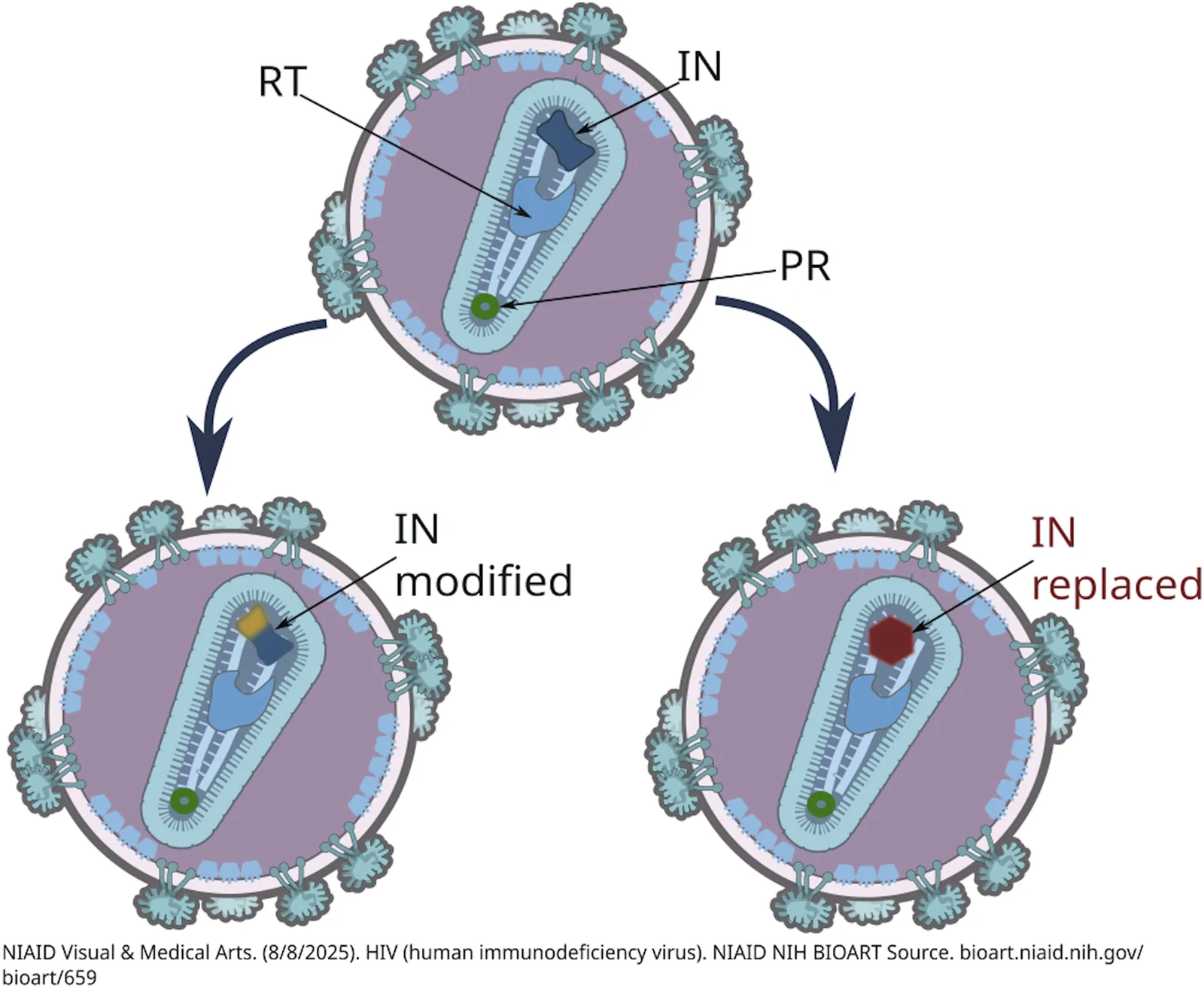
Examples of retrovirus-based hybrid viral vectors for modulation of the transgene integration profile. The upper panel shows the original virion structure with key enzymes: reverse transcriptase (RT), integrase (IN), and protease (PR). The lower-left panel illustrates various approaches involving a modified integrase, while the lower-right panel shows hybrid systems with the native integrase completely replaced, where alternative molecular mechanisms mediate transgene insertion according to their own specific integration patterns. The figure was assembled using vector artwork from the NIH BioArt Source repository: NIAID Visual and Medical Arts. (2025). HIV (human immunodeficiency virus), BIOART-000659. Available at: https://bioart.niaid.nih.gov/bioart/659.

Early efforts focused on retargeting integration by fusing integrase with sequence-specific DNA-binding domains recognizing short target motifs. Chimeric constructs based on λ-repressor or LexA domains (Bushman, 1994; Goulaouic and Chow, 1996; Katz et al., 1996) and Vpr-mediated delivery systems were able to incorporate modified integrase into viral particles; however, the requirement for multimerization of these DNA-binding domains (Weiss et al., 1987; Zhang et al., 2010), together with suboptimal intasome compatibility, resulted only in weak and poorly reproducible shifts in integration profiles, without detectable enrichment at defined target sites (Holmes-Son and Chow, 2000; Holmes-Son and Chow, 2002).

Subsequent strategies employed monomeric and engineered DNA-binding platforms. Zif268–integrase fusions produced only limited retargeting and were associated with reduced infectivity (Bushman and Miller, 1997), whereas more advanced polydactyl zinc-finger systems (E2C) enabled a measurable increase in integration near a unique target site in the erbB-2 locus (up to ∼10-fold enrichment locally), but at the cost of markedly reduced overall integration efficiency and infectivity (∼11–24% of wild-type levels) (Tan et al., 2004; Tan et al., 2006; Su et al., 2009).

A similar pattern was observed for integrase fusions with homing endonucleases such as I-PpoI (Schenkwein et al., 2010; Schenkwein et al., 2013). Despite enrichment of integration within rDNA loci (from ∼0.1% to ∼2–3%), effects remained modest and were strongly influenced by protein multimerization constraints and structural compatibility within the intasome. *In vivo* and engineered variants further confirmed only limited improvements (up to ∼3–4% target-associated integration) accompanied by substantial reductions in vector titer and efficiency (Nousiainen et al., 2023; Turkki et al., 2014). In addition, nuclease activity of I-PpoI induced pronounced cytotoxicity due to rDNA-associated double-strand breaks, while catalytically inactive variants reduced toxicity but did not improve targeting precision beyond background levels.

A distinct class of approaches emerged in γ-retroviral systems aiming not at absolute site-specific targeting but at reshaping global integration bias. Modifications of the MLV integrase C-terminal domain, including W390A substitutions disrupting BET protein interactions, resulted in redistribution of integration away from transcription start sites and CpG islands (Ashkar et al., 2014; Ashkar et al., 2017). Additional chromodomain fusions (e.g., CBX1) further reduced TSS-proximal integration to ∼5% of events. However, *in vivo* studies demonstrated that even pronounced shifts in integration landscapes did not eliminate oncogenic risk, as insertional mutagenesis was redistributed rather than removed, with alternative oncogenic targets (e.g., Notch1, Ppp1r16b) observed (Nombela et al., 2022).

Overall, these studies indicate that integrase engineering can modulate integration preferences to a limited extent, but is generally constrained by structural compatibility of modified intasomes and by trade-offs between targeting specificity and overall vector efficiency.

### 3.3 Hybrid HSV-based vectors

Among the viruses used for vector development, herpesviruses - predominantly herpes simplex viruses (HSV-1) - are characterized by one of the largest genome sizes, on the order of ∼152 kilobases (Macdonald et al., 2012; Smith et al., 2014). This feature makes them particularly attractive as viral vector platforms, since, when amplicon-based systems are used, they enable packaging of cargo of comparable size. “Amplicon” systems are replication-defective vectors consisting of plasmid-like circular DNA constructs containing minimal cis-acting herpesvirus elements: the origin of replication (ori) and the packaging signal (pac). In the presence of a helper system, amplicon DNA replicates via a rolling-circle mechanism, forming tandem linear concatemers that are “processed” into fragments that can be packaged into the viral capsid according to the permissible upper limit of packaged genome length. Their advantages include, in addition to high carrying capacity, a relatively broad tropism encompassing, for example, post-mitotic neurons, as well as comparatively low cytotoxicity and, due to the absence of viral gene expression, reduced immunogenicity (compared to replication-competent HSV vectors) (de Silva and Bowers, 2009; Epstein, 2009; Fernández-Frías et al., 2020; Le Hars et al., 2025).

However, the disadvantages – apart from production complexity and the risk of contamination associated with helper virus use – are predominantly context-dependent, and at various times attempts have been made to “address” them through the development of hybrid vectors based on herpes simplex viruses (Figure 3).

**FIGURE 3:**
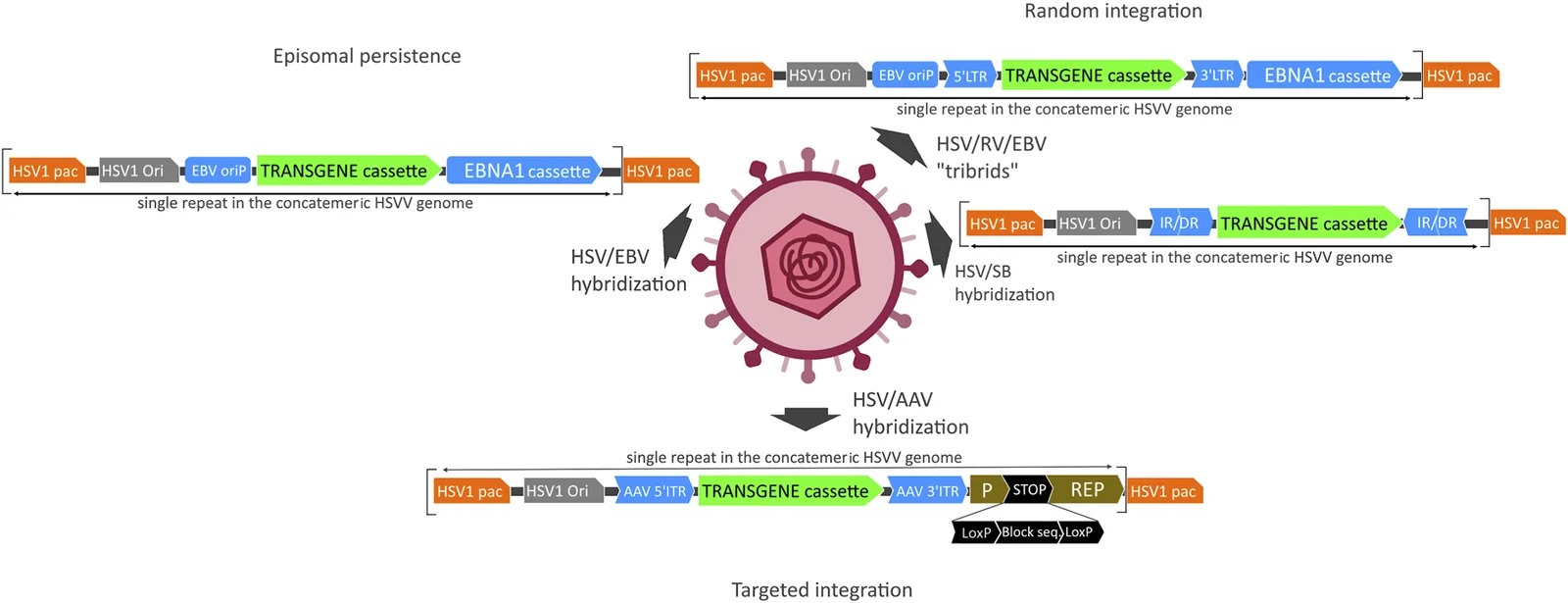
Examples of herpes simplex virus type 1 (HSV-1)-based hybrid systems for episomal persistence, random, and targeted integration. Episomal persistence: a single repeat within the concatemeric genome (here and below, diagrams show an individual monomer from multiple sequentially linked copies) of the HSV/EBV hybrid with EBV oriP and EBNA1 elements for extrachromosomal maintenance. Random integration: HSV/RV/EBV “tribrids” with retroviral 5′/3′ LTRs and EBV components, as well as HSV/SB hybrids with terminal IR/DR repeats for Sleeping Beauty transposition. Targeted integration: HSV/AAV hybrid with a transgene cassette flanked by AAV 5'/3′ ITRs and a rep gene, whose expression is initially blocked by a stop sequence between Cre recombinase recognition sites (LoxP) and is activated only in cells with Cre recombinase, which excises this blocking element. Created with BioRender.com.

#### 3.3.1 Integration of HSV-based vectors

One obvious limitation is the episomal persistence of amplicon vectors in host cells and, consequently, their limited duration of maintenance, particularly in dividing cells, from which they are eliminated relatively quickly (de Silva and Bowers, 2009; Le Hars et al., 2025). Given their large size and the presence of an envelope with surface glycoproteins (glycoproteins B, H/L, C, D, E/I, etc.), repeated administration of such vectors may be challenging. Therefore, the possibility of integration into the host genome could provide a significant advantage in certain applications.

##### 3.3.1.1 HSV/retroviral hybrids

The primary candidate for serving as a donor of integrase activity is always retroviral or lentiviral systems. In this context, such studies already emerged in the late 1990s. However, the field did not progress significantly beyond the early 2000s. Nevertheless, several generations of such vectors were developed. The first generation served primarily as a proof of concept. HSV-1 amplicons were used as transient carriers of trans-acting retroviral genes (gag/pol/env), enabling *in situ* “rescue” of already integrated defective retroviral proviruses and the production of infectious retroviral particles without the transfer of an integration-competent genome Herpesvirus/retrovirus chimeric vector (Savard et al., 1997; Parr et al., 1999).

In second-generation chimeric HSV-1 amplicon vectors, a more integrated and functionally coupled architecture was implemented. A single amplicon system combines both retroviral trans- and cis-cassettes (the Gag/Pol/Env expression cassette and a retroviral vector cassette carrying a therapeutic or marker gene), as well as additional elements derived from other viruses aimed at improving stability and prolonging expression (Sena-Esteves et al., 1999). This enables their coordinated expression within the same cell following a single infection.

Functionally, such systems were shown to convert infected cells into transient retrovirus-producing factories after a single-step transduction, enabling subsequent generation of infectious retroviral particles and secondary transduction of surrounding cells.

The use of helper-free amplicon production systems substantially reduced contamination risk and improved the reproducibility of retroviral production in infected cells. Additional incorporation of EBV elements, such as oriP and EBNA1, provided partial episomal stabilization of the amplicon genome in the nucleus, leading to prolonged expression of retroviral components and increased overall system productivity (Wang and Vos, 1996). In some constructs, LTRs and associated packaging-related regulatory elements within the vector cassette were also modified to modulate transcriptional and packaging activity.

High efficiency of the vector was reported even in cell lines relatively resistant to retroviral infection, as well as sustained and stable transgene expression in tumor cell models for at least 1 month. In several models, dose-dependent production of retroviral particles and prolonged maintenance of transgene activity over several weeks were observed (Sena-Esteves et al., 1999; Felipe et al., 2001).

##### 3.3.1.2 HSV/SB hybrids

The HSV-1/Sleeping Beauty (SB) system is a hybrid vector platform based on HSV amplicons enabling stable genomic integration of transgenes via transposon-mediated insertion. It is built on a bipartite design, in which a transgene cassette is flanked by inverted/direct repeats (IR/DR), while transposase is provided in trans (to prevent premature transposase activity in producer cells, which can excise the transgene from amplicon concatemers and reduce vector yield, and to mitigate SB overproduction inhibition (Bowers et al., 2006; de Silva et al., 2010a; de Silva et al., 2010b)) under an HSV immediate-early promoter (Bowers et al., 2006). *In vivo* studies, including *in utero* delivery into the ventricular system of embryonic mouse brain, demonstrated long-term transgene expression mediated by stable integration in both proliferating and postmitotic neurons, with up to ∼25-fold higher expression compared to conventional HSV amplicons *in vitro* (Bowers et al., 2006)). Preferential transduction of Sox1-positive neuroepithelial progenitors was observed, consistent with HSV-1 tropism, with subsequent differentiation of these cells while maintaining integrated cargo (Peterson et al., 2007). Although hyperactive SB variants such as HSB5 show increased catalytic activity *in vitro*, this does not translate into improved *in vivo* performance, where functional outcomes converge across variants in neural tissue (Tamin et al., 2009; Mirow et al., 2021).

Overall, the HSV/SB platform combines high HSV amplicon cargo capacity (∼150 kb) with SB-mediated stable expression, with largely random integration patterns reflecting the intrinsic properties of SB transposition, alongside constraints associated with dual-component delivery, particularly *in vivo* (de Silva and Bowers, 2011).

##### 3.3.1.3 HSV/AAV hybrids

Another virus that is always considered in the context of providing integrative activity is AAV, which has a clear advantage over retroviruses, if not in integration efficiency, then in its pronounced bias toward specific integration hotspots; for AAV2, whose elements are most commonly used, this is AAVS1 on chromosome 19 within the PPP1R12C gene. Indeed, hybrid systems involving HSV-1, which contributes high packaging capacity - a parameter in which rAAV is particularly limited - and AAV were developed based on amplicon vectors. The main body of work represents a sequential development of a single technological line formed through close cooperation between the laboratories of Xandra Breakefield (MGH/Harvard) and Cornel Fraefel (University of Zurich). The underlying concept was to incorporate into the amplicon vector, in essence, an rAAV genome (a cargo flanked by AAV ITRs).

In early proof-of-principle studies (Johnston et al., 1997), a standard HSV-1 amplicon was used, containing the elements required for its production - HSV replication origin oriS and packaging signal pac - into which a transgene expression cassette (lacZ under the CMV IE1 promoter) flanked by AAV ITR elements was inserted, as well as (in some constructs) an expression cassette for the AAV Rep gene. It was shown that in the presence of Rep, transgene expression in dividing human glioma cells was maintained for more than 2 weeks, whereas classical HSV amplicons exhibited rapid loss of expression.

Later, the mechanism of directed integration of the ITR-flanked cassette was confirmed, predominantly into the AAVS1 locus, in the presence of Rep68/78 supplied in trans, along with the functional role of Rep as a key factor in this process (Heister et al., 2002; Wang et al., 2002; Oehmig et al., 2007; Bakowska et al., 2003). A fundamental trade-off between integration efficiency and vector production due to the known toxicity of Rep expression products in cells, particularly in producer cells of hybrid vectors (as soon as expression in target cells is transient) was also highlighted (Glauser et al., 2006).

This limitation was addressed through regulated Rep expression, which had to be suppressed in producer cells. Reactivation of expression in target cells was achieved, for example, via Cre/loxP-dependent recombination that excised a “blocker” of Rep expression (in this case, a fragment of the neomycin phosphotransferase gene, neoR, flanked by loxP sites in direct orientation) in the presence of co-transduced Cre recombinase. Application of the Cre/loxP switch enabled an increase in packaged genome titers from ∼10^6^ to ∼10^8^ vg/mL, while infectious titers also increased by two orders of magnitude - from ∼2 × 10^5^ to ∼2 × 10^7^ TU/mL - bringing them to levels comparable with standard HSV-1 amplicon vectors (Liu et al., 2006). Stable transduction reached approximately ∼20% under optimal conditions.

The ability to integrate large DNA cargos exceeding 100 kb - demonstrated using a full-length β-galactosidase genomic locus including its regulatory elements - was confirmed as one of the intended design features of the system (Oehmig et al., 2007). An important observation was that large inserts do not alter the core Rep/ITR-mediated integration mechanism, although frequency of correctly formed preintegration units can be reduced, likely due to independent effects: reduced efficiency of HSV amplicon replication/packaging and, separately, reduced efficiency of Cre/loxP excision required for formation of the integrable unit.

Practically relevant results were demonstrated by Cortés et al., in 2008 (Cortés et al., 2008) using the functionally important ATM gene. In particular, restoration of the cellular radiation response was shown, confirming functional expression of the integrated gene, and integration into hotspot loci was assessed.

#### 3.3.2 Hybrid HSV-based viral vectors without integration

An alternative rationale for developing HSV-based hybrid vector systems, beyond enabling integration, is the stabilization of episomal persistence after transduction. Early efforts of this type date back to the late 1990s. In particular, in the work by Wang S et al. (Wang et al., 1997), a modified HSV-1 amplicon system (pHE) was described, in which the standard amplicon genome (containing HSV oriS and pac signals) was supplemented with elements derived from Epstein–Barr virus - namely oriP and the EBNA-1 gene under the control of a heterologous CMV IE promoter - inserted directly into the amplicon genome. Stable episomal maintenance of the vector DNA was thereby achieved through EBV-dependent replication and segregation. This design also enabled pre-selection of producer cells prior to packaging, resulting in increased amplicon yields. Importantly, the system retained the high cargo capacity characteristic of HSV amplicons (with inserts of approximately ∼20 kb demonstrated in this study) and supported efficient expression both *in vitro* and *in vivo*. Limitations of the system included the inherent dependence on helper virus for packaging, with the associated risk of helper contamination, as well as potential biological effects related to sustained EBNA-1 expression during long-term persistence.

Subsequent studies confirmed more sustained transgene expression in hepatocytes *in vivo* compared to conventional HSV amplicons (although gradual decline over time persisted); similarly, these hybrid vectors confer enhanced transgene expression in primary human tumors and human bone marrow-derived mesenchymal stem cells. Enhanced expression levels in primary human tumors and mesenchymal stem cells were demonstrated, highlighting improved performance in otherwise poorly transduced cell types (Müller et al., 2005; Sia et al., 2010).

### 3.4 Representative rAAV-based hybrid systems

AAV has been employed in a vast number of hybrid systems. Here, however, we will not delve into examples of chimerization within the Parvoviridae family involving capsid manipulations, primarily aimed at modulating tropism (essentially various forms of pseudotyping), which appear to be the most common for parvoviral vectors in general and, particularly, for rAAV. Instead, our focus is on hybridization with representatives of other viral groups outside the parvoviruses.

#### 3.4.1 AAV/phage hybrid systems

Among the more conceptually nontrivial approaches are systems combining AAV with bacteriophages (AAVP and related constructs) (Figure 4a). Despite the seemingly counterintuitive choice of a prokaryotic viral platform, such architectures follow a clear engineering rationale: the phage capsid is used as a highly modifiable and functionally minimal scaffold lacking predefined mechanisms for tropism, intracellular trafficking, and nuclear delivery. Consequently, all these properties can be imposed artificially – for example, via ligands such as RGD4C targeting αV integrins, whereas the AAV component (an ITR-flanked cassette) governs the fate of the delivered genome, including its stabilization as head-to-tail concatemers and the maintenance of transgene expression.

**FIGURE 4:**
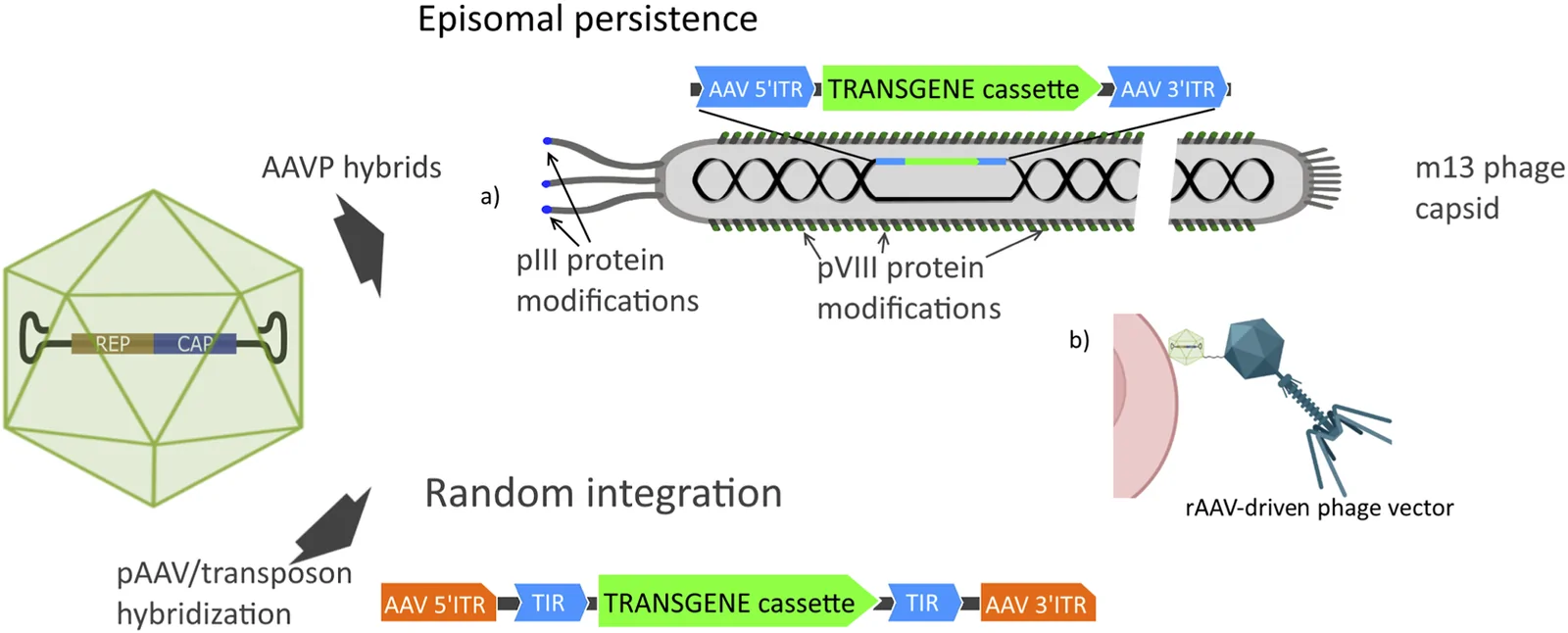
Selected examples of recombinant adeno-associated virus (rAAV)-based hybrid systems for episomal persistence and genomic integration. Episomal persistence: **(a)** genome structure of hybrid vectors involving filamentous m13 phages with pIII and pVIII protein modifications; **(b)** dual-virion hybrid vector based on bacteriophage T4 and a chemically linked rAAV acting as a “driver” to mediate receptor binding and endocytosis. Random integration: section demonstrates a generalized structure of randomly integrating hybrid vectors based on rAAV and various types of transposons. Created with BioRender.com.

In case of AAVP, it is a single composite particle in which the rAAV genome is physically embedded into the genome of a filamentous bacteriophage (typically an M13-derived fd-tet backbone), while the capsid is functionally retargeted through peptide display on coat proteins (most commonly pIII and/or pVIII). In this configuration, the phage acts as an integrated carrier: it mediates receptor-specific binding and cellular uptake, and remains the structural vehicle up to the point of intracellular uncoating and release of the packaged DNA. In early studies, the AAVP vector genome is a circular single-stranded DNA molecule in which the complete genetic apparatus of the fd-tet bacteriophage is preserved, including all structural and regulatory genes (gI–gXI), as well as the tetracycline resistance cassette. The AAV component, consisting of an expression cassette (promoter, transgene, and polyadenylation signal), was inserted into an intergenic region (IG) of the phage genome (Hajitou et al., 2006; Hajitou et al., 2007). The presence of AAV ITRs within the phage vector is associated with more prolonged and stable transgene expression, which correlates with the formation of concatemeric forms of the delivered AAV cassette in the nucleus, likely arising through recombination between ITR elements and, presumably, episomal persistence of such forms, although their molecular nature remains not fully defined.

Receptor-binding specificity of the hybrid AAVP is achieved through the display of peptide ligands on the surface of the phage capsid, introduced by genetic modification of the minor coat protein pIII (and, in some constructs, the major coat protein pVIII), most commonly via insertion of the cyclic RGD motif (RGD4C) in a manner that enables its surface presentation. It has been shown *in vivo* that RGD4C-decorated AAVP, upon systemic administration, selectively accumulate in tumor tissue via specific interactions with αv integrins expressed on tumor cells, predominantly on tumor-associated vascular endothelium, and mediate receptor-dependent entry into target cells via clathrin- and dynamin-dependent endocytosis, thereby functioning as tumor-targeted vectors (Hajitou et al., 2008; Tandle et al., 2009; Stoneham et al., 2012; Kia et al., 2012; Przystal et al., 2019; Staquicini et al., 2020).

In contrast to many hybrid viral vector strategies, this approach has not yet fallen into oblivion. For example, the study by Smith et al. demonstrated the possibility of extending the AAVP platform by replacing the RGD-based targeting strategy with an alternative receptor-specific homing mechanism. In this construct, the phage capsid was functionalized with the octreotide peptide, enabling specific binding to the somatostatin receptor SSTR2, which is expressed on pancreatic neuroendocrine tumor cells. This modification allowed receptor-mediated accumulation in tumor tissue *in vivo* and efficient delivery of the therapeutic TNF gene, which was accompanied by a pronounced antitumor effect in the experimental model. A comprehensive strategy was later implemented by Suwan et al. (Suwan et al., 2019), who, through modification of the major capsid protein pVIII and the combined display of multiple peptide domains, achieved simultaneous implementation of targeting (RGD4C), reduction of non-specific adsorption and interactions with plasma components (including AKAS-mediated reduction of the protein corona), as well as enhancement of endosomal escape via a histidine-containing H5WYG motif. Collectively, these modifications led to a substantial increase in transgene expression and expanded the possibilities for further multifunctional engineering of these hybrid vector systems.

Not all phage/AAV hybrid constructs were developed for oncological targeting. For example, in 2018, Khalili et al. (Khalili et al., 2018) proposed a hybrid vector based on an M13 bacteriophage, into which transgene expression cassette flanked by AAV ITRs was incorporated. In this case, surface modification with the TAT peptide enhanced cellular internalization efficiency without conferring pronounced receptor-specific tropism.

Recently, hybrid constructs based on bacteriophage T4 have been developed as dual-virion complexes, employing a concept that differs significantly from earlier designs (Figure 4b). This bionanotechnology platform combines the substantial payload capacity of bacteriophage T4 (up to 170 kb) with the transduction capability of adeno-associated virus (AAV) for delivery into mammalian cells. Assembly of the hybrid vector involves non-covalent attachment of the biotinylated recombinant rAAV2 virions, encoding reporter protein, to the T4 surface via avidin-biotin bridges and Soc/Hoc auxiliary proteins. In this architecture, rAAV2 acts as a “driver,” mediating receptor binding, initiating receptor-mediated endocytosis, and facilitating the endosomal escape of the entire complex into the cytosol for trafficking to the nucleus. By utilizing host nuclear import pathways, the complex delivers large genetic cassettes and functional proteins (e.g., dystrophin or full-length antibodies) directly into the nucleus. Stable cargo expression was observed for at least 60 days. This strategy achieves a 40,000-fold increase in delivery efficiency compared to native T4, representing a functional transition from the phage’s natural inertness in mammalian cells to therapeutically significant absolute expression levels. Its high therapeutic potential has been demonstrated by providing complete protection against pneumonic plague in murine vaccine models (Zhu et al., 2019; Zhu et al., 2020).

#### 3.4.2 Integrating rAAV/transposon systems

Among the active and still ongoing directions is the development of integrating vectors in which delivery is mediated by well-characterized recombinant adeno-associated viruses (rAAVs), which are efficient and broadly applicable across a wide range of cell types, while the intracellular fate of the cargo is determined by the presence of transposon elements (most commonly–of Sleeping Beauty or piggyBac transposons) (Figure 4). The idea underlying this approach is to overcome a key limitation of recombinant AAVs - transient transgene expression due to gradual dilution of episomally persisting vector genomes during cell division. The theoretical foundation for this strategy was established in studies from the 2000s–2010s. These works demonstrated the practical feasibility of using rAAV for delivery of an SB transposon, followed by genomic integration mediated by hyperactive transposase variants, such as HSB5 (described as 10-fold more active than the wild-type enzyme) and, subsequently, SB100/SB100X (up to 100-fold more active than the wild-type enzyme), supplied in trans. It was also shown that the characteristic rAAV propensity to form concatemers and persist as circular episomes is sufficient to support transposase activity. Use of SB100X transposase in a dual-rAAV system increased the number of stable integration events by approximately 10-fold compared to an inactive transposase control; up to ∼98% of integrations were SB-mediated, and analysis of ∼1,800 integration sites revealed an approximately random distribution, with ∼49% located within gene bodies (predominantly - intronic) (Müther et al., 2009b; Zhang et al., 2013).

A number of more recent studies (Cunningham et al., 2024; Ye et al., 2023; Zakas et al., 2024; La et al., 2020) have extended this strategy for overcoming loss of episomal rAAV DNA by incorporating transposon systems that mediate stable genomic integration, resulting in a mosaic population of cells stably expressing the transgene at levels sufficient to achieve therapeutic or other desired effects in the context of tissue growth. It has been demonstrated that the rAAV/Sleeping Beauty (SB100X) system can sustain long-term expression and functional correction of metabolic defects in the rapidly growing neonatal liver, where standard rAAV vectors are rapidly diluted. Finally, Zakas et al., in 2024 proposed delivering the transposase as transiently translated mRNA encapsulated in lipid nanoparticles (LNPs), thereby temporally separating transposase expression from integration activity, reducing the risk of transposon remobilization while simultaneously improving system efficiency, including enhanced dose–response performance in preclinical models, including non-human primates.

Among the obvious limitations of the system are the limited packaging capacity associated with the rAAV platform as the delivery vehicle, as well as increased complexity due to its two-component nature, which is particularly relevant in the context of *in vivo* applications. In addition, transposon-mediated integration is inherently largely random, which is not always a desirable outcome. However, this limitation is potentially addressable. For example, as early as 2012, Ammar et al. described retargeting of Sleeping Beauty, piggyBac, and Tol2 transposase activity (Ammar et al., 2012). By generating fusion proteins between these transposases and DNA-binding domains derived from AAV Rep, the authors achieved a ∼15-fold increase (for SB transposase) in targeted integration frequency at a safe harbor locus (in a plasmid-based model). Attempts to retarget piggyBac and Tol2 were less efficient and, in some cases, resulted in substantial reductions in transposase activity.

### 3.5 Baculovirus hybrids

Baculovirus genomes are large circular double-stranded DNA molecules typically ranging from ∼80 to 180 kb, with commonly used alphabaculoviruses such as Autographa californica multiple nucleopolyhedrovirus (AcMNPV) being ∼130 kb in size, and engineered baculovirus expression systems generally supporting insertions on the order of up to ∼30–40 kb. These viruses are insect-specific and extensively used in the baculovirus expression vector system (BEVS) for high-level recombinant protein production, and under certain experimental conditions they can transduce mammalian cells without productive replication, thereby functioning as transient gene delivery vectors (Hu, 2006; Airenne et al., 2013; Felberbaum, 2015).

The main limitations of baculovirus-based systems in gene therapy applications include their strict insect host tropism and, thus, - the requirement for artificial envelope modification (pseudotyping, e.g. with VSV-G) to achieve efficient entry into mammalian cells, the absence of native mechanisms for stable episomal maintenance or genomic integration resulting in predominantly transient transgene expression, the induction of innate and adaptive immune responses upon *in vivo* administration which limits systemic use and repeat dosing, and their comparatively limited clinical and regulatory maturation relative to established platforms such as AAV and adenoviral vectors, which together result in their primary application being in recombinant protein and viral vector manufacturing rather than as standalone *in vivo* gene delivery systems (Figure 5).

**FIGURE 5:**
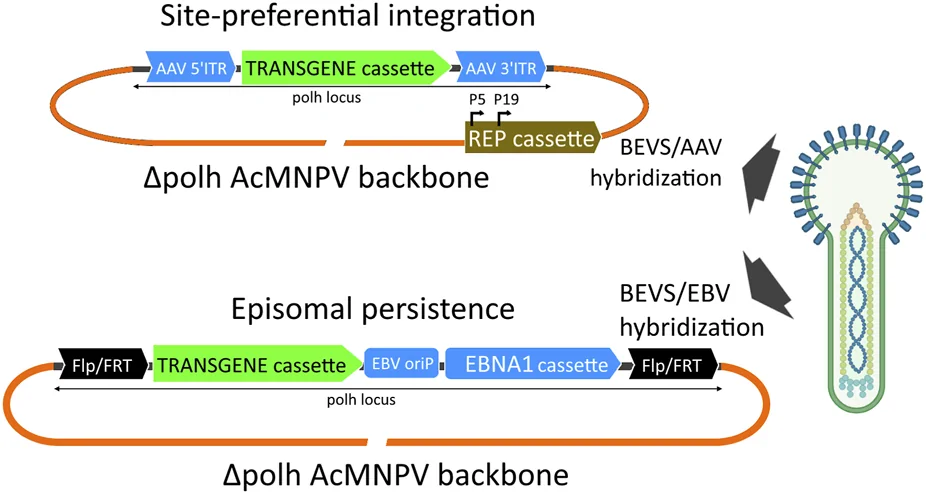
Examples of baculovirus expression vector system (BEVS)-based hybrid systems for site-preferential integration and episomal persistence. Site-preferential integration: a system based on the AcMNPV (Autographa californica multiple nucleopolyhedrovirus) backbone with a deleted polyhedrin gene (Δpolh), containing a transgene expression cassette flanked by AAV 5′/3′ inverted terminal repeats (ITR) and a rep gene cassette driven by P5 and P19 promoters within the polh locus for targeted integration. Episomal persistence: a construct within the polh locus of the Δpolh AcMNPV backbone, including the target transgene and EBV oriP/EBNA1 elements for extrachromosomal maintenance, flanked by Flp/FRT sites for subsequent excision and circularization. Created with BioRender.com.

Nevertheless, there have been attempts to bypass some of the aforementioned limitations of baculovirus vectors (BVVs) using hybrid viral systems. As early as 1998, Palombo et al. proposed such a system based on an almost full-length genome of the baculovirus *Autographa californica multiple nucleopolyhedrovirus* (AcMNPV), approximately 130 kb in size, in which the complete DNA replication and nucleocapsid assembly machinery is preserved, while the polyhedrin (polh) gene - non-essential for replication in cell culture - was deleted (Palombo et al., 1998). AAV components were inserted into the polh locus and included a transgene cassette flanked by inverted terminal repeats (ITRs), as well as a rep expression cassette driven by its native p5 and p19 promoters in antisense orientation relative to the baculoviral polyhedrin promoter. This configuration suppresses Rep expression during vector production and prevents premature excision of the ITR-flanked cassette from the baculoviral genome. The presence of rep in the hybrid vector resulted in a 10–50-fold increase in the number of stable cellular clones compared to rep-deficient constructs. The authors also reported site-specific integration of the ITR-flanked cassette into the AAVS1 locus on human chromosome 19 in approximately 36%–41% of cases.

In a later study, Wang et al. adapted baculovirus–AAV hybrid (HyV) technology for neurobiological applications, including models relevant to Parkinson’s and Alzheimer’s disease. The vector architecture was largely similar to that described above. The use of tissue-specific promoters (neuronal CMV enhancer/PDGF and astrocytic CMV enhancer/GFAP) restricted transgene expression to defined CNS cell types. It was further shown that sustained expression was observed primarily in human cell lines (HCN-2, U251), associated with integration into the AAVS1 locus on chromosome 19, whereas in rodent cell lines (PC12, C6), which lack a compatible integration site, expression decayed at a rate comparable to that of standard baculovirus-mediated delivery (Wang, 2008).

The system described by Lo et al. (Lo et al., 2009) employs FLP recombinase delivered via a helper baculovirus vector (BacFLP) to excise a genetic cassette from the baculoviral genome, converting it into a circular episome inside the target cell. Owing to the presence of Epstein–Barr virus-derived oriP/EBNA1 elements, this episome is autonomously replicated and maintained in daughter cells for more than 2 months, while the baculoviral backbone is rapidly degraded. Functional relevance was demonstrated in mesenchymal stem cells, where sustained production of BMP-2 efficiently promoted osteogenesis. The authors emphasized the safety of the approach, noting that the baculoviral backbone is eliminated without residual genetic material, thereby reducing the risk of insertional mutagenesis (Luo et al., 2012).

## 4 Conclusion

Despite decades of development of viral and hybrid vector systems, their evolution remains constrained by fundamental biological and technological barriers arising from mismatches between optimal solutions for delivery, intracellular processing, and expression of genetic material. The concept of hybridization logically follows from the modular organization of viral functions, implying the possibility of redistributing them across different platforms. However, in practice, functional decomposition of viral systems rarely leads to linear improvements in performance, since the integration of heterologous modules is accompanied by epistatic interactions and disruption of coordination across vector life-cycle stages.

Historically proposed complex multicomponent hybrid constructs have so far seen limited clinical translation, as indicated by the relatively small body of evidence for their clinical use, despite decades of development. Potential contributing factors include reduced efficiency associated with increasing complexity of multistep delivery systems, incompatibility between packaging and replication mechanisms of different viral platforms, and constraints related to cytotoxicity of individual components and the need for coordinated expression of multiple genetic modules. An additional limiting factor is the immunological burden arising from the combination of viral elements of different origins, which restricts both efficacy and the possibility of repeated administration.

In parallel, optimized “monoplatform” systems have undergone substantial development, most prominently adeno-associated viral (AAV) and lentiviral vectors, as well as non-viral delivery platforms including lipid nanoparticles (LNPs) and polymer-based carriers. Alternative gene delivery and expression systems, such as engineered AAV capsids with enhanced tropism and production properties, lentiviral vectors with modified integration specificity, and LNP platforms that have become central for mRNA and genome-editing delivery, have further expanded the available toolkit. In these systems, directed evolution of capsids, engineering of regulatory elements, and optimization of manufacturing processes have substantially expanded therapeutic potential without the need to adopt complex chimeric architectures. As a result, a significant fraction of tasks that previously motivated the development of hybrid constructs has been partially redirected toward improving existing platforms, leading to a shift of hybrid strategies toward specialized solutions aimed at overcoming specific functional limitations rather than achieving universal optimization of delivery.

Nevertheless, monoplatform systems remain subject to intrinsic trade-offs regarding key parameters such as transduction efficiency, payload capacity, tissue specificity, immunogenicity, and duration of expression. While non-viral approaches have further strengthened this alternative trajectory due to their scalability and reduced immunogenicity, they still retain limitations related to extracellular and intracellular targeting, efficiency of endosomal escape, and the absence of native mechanisms for nuclear delivery or genomic integration. The persistence of these structural gaps between required functional stages of delivery underlies the continued relevance of hybrid systems, which are inherently designed to redistribute functions among initially poorly compatible yet functionally complementary biological modules, thereby enabling emergent properties that cannot be achieved within a single evolutionarily or technologically constrained platform (Table 2). In our view, the most promising hybrid strategies are currently those in which the combination of modules provides a reproducible improvement of specific delivery stages without critically compromising overall biological compatibility. These include systems combining viral delivery with transposon-based integration, constructs enabling episomal persistence through EBV or AAV elements, as well as phage- and baculovirus-based platforms used to extend cargo capacity and modify tropism.

**TABLE 2:** Comparative characteristics of selected hybrid gene-delivery platforms.

Platform	Cargo capacity	Persistence/integration behavior	Immunological burden	Production/use complexity
Pseudotyped retroviral/lentiviral vectors — retroviral or lentiviral particles displaying heterologous envelopes such as VSV-G, RV-G, alphavirus E1/E2, paramyxovirus H/F or G/F, GP64, LCMV-G, influenza HA/NA, Ebola GP, coronavirus S, dengue or HCV glycoproteins (Wool-Lewis and Bates, 1998; Reiser, 2000; Hsu et al., 2003; Schauber et al., 2004; Giroglou et al., 2004; McKay et al., 2006; Dylla et al., 2011; Duvergé and Negroni, 2020; Yoder et al., 2021; Dautzenberg et al., 2021)	Moderate, Determined by backbone; typically, approximately 7–9 kb	Stable proviral predominantly non-targeted integration with integration-site preferences (MLV biased toward regulatory regions, LV toward gene bodies, etc.)	Variable to high; depends strongly on the heterologous envelope, pre-existing antibodies, complement sensitivity, and administration route	Moderate to very high; VSV-G is relatively standardized, whereas many alternative envelopes reduce titre and require extensive optimization
AdV/AAV — adenoviral vector carrying an AAV ITR-flanked cassette (Fisher et al., 1996; Lieber et al., 1999; Recchia et al., 1999; Shayakhmetov et al., 2002; Ueno et al., 2000; Recchia et al., 2004)	Limited by the AdV capsid packaging capacity (up to 37 kb for HDAd)	Episomal persistence and rare nonspecific integration in the absence of Rep; modest frequency integration (preferably within the AAVS1 site) with REP provided cis or trans	High, primarily due to the AdV capsid; additional burden from AAV components and Rep	High; may require Rep regulation, helper-dependent AdV, or co-delivery of additional components
AdV/EBV — adenoviral vector containing EBV oriP/EBNA1 elements (Tan et al., 1999; Gil et al., 2010)	AdV-specific high; EBV elements take space from the overall capacity (∼2–3 kb for oriP/EBNA1)	EBV-mediated episomal maintenance; persistence for weeks to ∼3–5 months in dividing-cell models, with longer persistence in non-dividing tissues *in vivo*	AdV-specific high; additional concerns related to sustained EBNA1 expression	Moderate to high; may involve Cre/LoxP processing (if used), helper Ad or plasmids, and dual-vector delivery
AdV/Sleeping Beauty — adenoviral delivery of an SB transposon cassette, optional - transposase gene (Yant et al., 2002; Boehme et al., 2016)	AdV-specific high; reduced by ∼2–4 kb for the SB cassette	Stable maintenance after SB-mediated transposition and predominantly random integration at TA dinucleotides	AdV-specific high; additional burden from transposase expression	High; may require co-delivery of donor and transposase, as well as Flp/FRT-mediated excision
AdV/LINE-1 — adenoviral delivery of a LINE-1 retrotransposon cassette (Soifer et al., 2001; Soifer and Kasahara, 2004; Kubo et al., 2006)	AdV-specific high; reduced by ∼6 kb for the LINE-1 cassette	Stable maintenance after target-primed rt-mediated retrotransposition with quasi-random integration	AdV-specific high; additional concerns arise from LINE-1 proteins	High; large L1 cassette, need to control L1 activity in producer cells to favor retrotransposition in target cells
AdV/PhiC31 — adenoviral vector delivering an attB-flanked cassette and PhiC31 integrase (Thorpe and Smith, 1998; Thor et al., 2000; Chalberg et al., 2006; Aneja et al., 2007)	AdV-specific high; reduced by ∼2 kb for PhiC31 if encoded in cis	Long-term expression after chromosomal integration into a limited set of pseudo-attP sites (not strictly site-specific)	AdV-specific high; potential additional burden from integrase expression	Moderate; may require a separate integrase-expressing vector for trans delivery
AdV/retroviral vectors — adenoviral delivery of retroviral cis-elements and, in some systems, packaging functions (Feng et al., 1997; Soifer et al., 2002; Kubo and Mitani, 2003; Picard-Maureau et al., 2004)	AdV-specific high; reduced by ∼3.8 kb for retroviral sequences (down to ∼1.5 kb for lentiviral)	Stable proviral persistence after non-targeted integration; integration-site preferences differ among retroviral groups, with MLV biased toward regulatory regions, LV toward gene bodies, and foamy-virus vectors showing comparatively weak integration-site bias	AdV-specific high; additional immunological burden depends on the retroviral component, with foamy-virus components generally considered relatively low in immunogenicity	High; Moderate to high; in some configurations, could have required Gag/Pol/Env expression, trans-complementation, and control of secondary particle formation in target cells. Additional genotoxic and biosafety concerns from retroviral components; foamy-virus components are generally considered low-risk
HSV/retrovirus — HSV amplicon carrying retroviral cis-elements and trans-acting packaging functions (Savard et al., 1997; Parr et al., 1999)	HSV-specific high; up to ∼150 kb	Transient production of secondary retroviral particles, followed by stable proviral (predominantly non-targeted) integration after secondary transduction	High; combined HSV and retroviral burden	High; requires coordinated expression of Gag/Pol/Env and, in some configurations, control of helper-virus contamination
HSV/retrovirus/EBV — HSV amplicon combining retroviral modules with EBV oriP/EBNA1 (Sena-Esteves et al., 1999; Wang and Vos, 1996; Hampl et al., 2003)	HSV-specific high; up to ∼150 kb; reduced by the retroviral and EBV modules	EBV elements support episomal replication and retention of the HSV amplicon in dividing cells; secondary retroviral particles provide stable, predominantly non-targeted integration	High; combined HSV, retroviral, and EBV components	Very high; requires coordinated expression of multiple viral components and, in some configurations, control of helper-virus contamination
HSV/Sleeping Beauty — HSV amplicon carrying an SB transposon cassette (Bowers et al., 2006; Peterson et al., 2007; de Silva et al., 2010b; de Silva et al., 2010a)	HSV-specific high	Stable transgene maintenance after SB-mediated transposition with predominantly random integration	Moderate to high; HSV is the principal immunological contributor	High; requires HSV amplicon production and separate transposase delivery
HSV/AAV — HSV amplicon carrying an AAV ITR-flanked cassette and regulated Rep (Johnston et al., 1997; Heister et al., 2002; Oehmig et al., 2007)	HSV-specific high	Rep/ITR-mediated chromosomal integration (moderate frequency, preferentially at the AAVS1 site), with episomal intermediates	Moderate; primarily attributable to the HSV-derived vector	High; may require Rep-associated cytotoxicity suppression during production and activation in target cells, in some approaches - through the Cre/loxP action
HSV/EBV — HSV amplicon containing EBV oriP/EBNA1 elements (Wang et al., 1997; Müller et al., 2005; Sia et al., 2010)	HSV-specific high	EBV-dependent episomal maintenance of the HSV amplicon; persistence for weeks to ∼3 months in dividing-cell models and prolonged expression for weeks to ∼1 year in in vivo models	Moderate to high; burden associated mainly with the HSV-derived vector and sustained EBNA1 expression	Moderate to high; helper-free systems reduce contamination risk but remain technically demanding
AAVP/M13 or fd-tet — filamentous phage carrying an AAV expression cassette with engineered pIII/pVIII targeting (Hajitou et al., 2006; Hajitou et al., 2007; Stoneham et al., 2012; Suwan et al., 2019; Jaroszewicz et al., 2022)	High - up to ∼12 kb additional cargo	AAV ITR-associated concatemer formation and predominantly episomal maintenance	Moderate to high; the phage particle is inherently immunogenic	Low to moderate; requires insertion of the cargo cassette into the established phage backbone and phage production
rAAV/T4 — T4 phage chemically or non-covalently associated with recombinant AAV particles (Zhu et al., 2019; Zhu et al., 2020)	Very high, T4 phage-specific: up to 170 kb	Mainly episomal persistence of the AAV-derived cargo	Moderate to high; two viral particles are combined	Very high; requires dual-virion assembly, chemical coupling, and control of complex stoichiometry
rAAV/transposon systems — rAAV delivering a transposon cassette, primarily Sleeping Beauty (Zhang et al., 2013; Ye et al., 2023; Cunningham et al., 2024; Zakas et al., 2024)	Low, limited by rAAV packaging capacity, approximately up to 4.5 kb	Stable persistence after transposase-mediated largely random genomic insertion	Low to moderate; AAV capsid dependent	May be high at usage stage - often requires dual-rAAV delivery or separate transposase delivery
BEVS/AAV — baculovirus carrying an AAV ITR-flanked cassette and Rep (Palombo et al., 1998; Hu, 2006; Wang, 2008; Lo et al., 2009; Airenne et al., 2013; Felberbaum, 2015)	High; up to ∼38 kb tested, potentially up to ∼130 kb	Rep/ITR-mediated AAVS1 preferring integration (in permissive cells) or prolonged maintenance	Low to moderate compared with AdV- or HSV-based systems	Moderate to high; insect-cell production is scalable, but Rep regulation and purification complicate the process
BEVS/EBV — baculovirus carrying EBV oriP/EBNA1 elements and an excisable cassette (Palombo et al., 1998; Hu, 2006; Wang, 2008; Lo et al., 2009; Kiyosue and Miwa, 2020)	High; baculovirus-specific, potentially up to ∼130 kb	FLP/FRT-mediated excision followed by EBV-dependent episomal maintenance	Low to moderate; baculovirus is nonpathogenic in humans, but EBNA1 and systemic administration remain relevant	Moderate; insect-cell production is scalable, but helper baculovirus and excision control are required

Finally, our observations suggest that viral vector hybridization is unlikely to represent a universal alternative to existing platforms and is more plausibly viewed as a targeted strategy for extending specific functional capabilities of established systems. Current developments favor functionally constrained combinations of modules rather than increasingly complex multicomponent architectures, with emphasis on maintaining a balance between performance, manufacturability, and biological compatibility.
